# Targeting PD-L1 for PCNS-DLBCL: from molecular effects to clinical translation

**DOI:** 10.3389/fimmu.2025.1647045

**Published:** 2025-09-26

**Authors:** Jiajia Cao, Shuzhen Xiong, Shuni Zhang, Ningning Yue, Chongyang Wu

**Affiliations:** Department of Hematology, The Second Hospital of Lanzhou University, Lanzhou, China

**Keywords:** PCNSL, PD-L1, biomarkers, immune checkpoint inhibitors, molecular targeted therapy

## Abstract

Primary central nervous system lymphoma (PCNSL) is a highly aggressive central nervous system lymphoma that has a high relapse rate and a 5-year survival rate of 30%-40% with conventional treatment. In about 95% of cases, Primary Central Nervous System Diffuse Large B-cell Lymphoma (PCNS-DLBCL) occurs. In some patients, the tumor microenvironment exhibited high levels of PD-L1, which may be linked to prognosis. The key mechanism for PD-L1 overexpression in EBV^-^ tumor cells is the amplification of the 9p24.1 copy number, with signaling pathways such as JAK2 and NF-κB possibly playing a role in this process. Immune checkpoint inhibitors (anti-PD-1/PD-L1 mAb), particularly combined with BTK inhibitors, show promise in relapsed/refractory PCNSL. Still, there is no universally accepted therapeutic consensus. The blood-brain barrier limits drug penetration, and the spatiotemporal heterogeneity of PD-L1 remains a challenge. This paper discusses the expression of PD-L1 in PCNS-DLBCL and its relationship to prognosis, the efficacy of anti-PD-1 mAb and other drugs, and possible predictive markers of efficacy to provide a basis for anti-PD-1/PD-L1 mAb therapy, and the future of targeted PD-L1 therapy to achieve a high remission rate and individualized immunotherapy for PCNSL patients.

## 1 Introduction

Primary central nervous system lymphoma (PCNSL) predominantly affects the brain parenchyma, leptomeninges, ocular structures, and spinal cord, and is classified as a subtype of non-Hodgkin’s lymphoma (NHL). Comprising roughly 3% of central nervous system (CNS) malignancies (1, 2). Approximately 90% of cases are pathologically categorized as diffuse large B-cell lymphoma (DLBCL), it is also known as PCNS-DLBCL (3). The existence of the blood-brain barrier (BBB) complicates the administration of most pharmaceuticals towards the tumor location, resulting in an extremely poor prognosis (4). In recent years, induction therapy utilizing rituximab in conjunction with alkylating agents and high-dose methotrexate (HD-MTX), succeeded by consolidation therapy comprising high-dose chemotherapy with thiotepa and autologous stem cell transplantation (ASCT), has markedly enhanced the prognosis for younger patients (5–7). Nonetheless, older individuals, particularly those over 70, demonstrate elevated incidence rates and frequently encounter difficulties in tolerating aggressive treatments such as high-dose chemotherapy or ASCT (8). As a result, their treatment outcomes are suboptimal, with 5-year survival rates between 30% and 40% (9). Consequently, optimizing therapeutic protocols for those with PCNSL in the elderly remains an essential priority in future clinical investigations.

Advances in delineating the genomic landscape and immune microenvironment of PCNSL have facilitated the advancement of individualized immunotherapies, such as Bruton’s tyrosine kinase (BTK) inhibitors (10), immune-modulating agents (11) and immune checkpoint inhibitors (ICIs) such as anti-PD-1 monoclonal antibody (anti-PD1 mAb) (12) have been attempted and applied in the treatment. They have achieved positive efficacy. The programmed cell death 1 receptor (PD-1) and its ligand PD-L1 are prominent focal points in oncology research. Membrane-type PD-L1 originating from tumor cells is known to engage with PD-1 on T cells, hence limiting T cell activation and diminishing toxicity. However, Atypical PD-L1, including cytoplasmic and cytosolic forms derived from tumor cells, frequently operates independently of the PD-1 pathway to exert immunosuppressive effects, enhance tumor proliferation, and regulate gene expression, thereby facilitating tumor growth and immune evasion (13). Solid tumor studies delineate dual PD-L1 regulatory mechanisms: Intrinsic pathways driven by 9p24.1 (the genomic locus of PD-L1) alterations (amplification/translocation) (14), oncogenic signaling (Ras-MEK (15), PI3K-Akt (16), JAK-STAT (17), and Abnormal alterations of cancer-related genes (C-MYC (18), CD58 (19, 20); Extrinsic pathways mediated by cytokine networks [IFN-γ (17), IL-10 (18)] within the immunosuppressive microenvironment. Previous investigations from our group demonstrated heightened PD-L1 levels in the tumor microenvironment (TME) of PCNS-DLBCL, showing significant association with clinical outcomes (21); A multicenter retrospective study (n=22) assessing the efficacy of nivolumab monotherapy in refractory/relapsed (R/R) PCNSL revealed that 41% of patients exhibited a favorable response to nivolumab, with a duration of response (DoR) surpassing 20 months (22). This suggests that the treatment strategy of inhibiting the PD-1/PD-L1 axis demonstrates better efficacy within PCNS-DLBCL, especially for R/R cases, and highlight its promise as a viable clinical option.

However, the limited research on PD-L1 protein expression and prognosis in PCNS-DLBCL, as well as on the efficacy of anti-PD-1/PD-L1 monoclonal antibodies and the identification of predictive biomarkers for treatment response. This paper will examine the expression levels of PD-L1 in PCNS-DLBCL, along with its prognostic implications, assess the efficacy of associated therapeutic agents such as anti-PD-1 mAb, identify potential predictive biomarkers of effectiveness, and explore future research avenues. Provide evidence and insights for PD-L1-targeted therapy in PCNSL (particularly in R/R patients).

## 2 Expression and function of PD-L1 protein in TME of PCNS-DLBCL

### 2.1 Expression of PD-L1 protein in TME of PCNS-DLBCL and its relationship with prognosis

PD-L1 protein expression is undetectable in normal brain biopsy specimens; therefore, PD-L1-positive cells identified in biopsy specimens from PCNSL patients are likely tumor cells or tumor associated immune cells (23), comprising tumor infiltrating lymphocytes (TILs), myeloid-derived suppressor cells (MDSCs), dendritic cells (DCs), as well as macrophages (24). Moreover, PD-L1 protein is found in TME and the peripheral blood immune system as a soluble or exosomal protein (sPD-L1, exoPD-L1), which engages with PD-1 and exhibits an immunomodulatory role (25). The research identified that PD-L1 and PD-1 upregulation commonly manifested in TME via immunohistochemical analysis of tumor specimens from 20 patients with PCNSL. PD-L1 was predominantly present on neoplastic cells and tumor-associated macrophages (TAMs). PD-1 was predominantly located in TILs and could also usually be present on TAMs, and is rarely expressed on tumor cells (26). The expression of these molecules may be associated with prognosis. summarized in **Table 1**. Different definitions about PD-1/PD-L1 positivity across studies resulted in discrepancies in positive expression rates and their prognostic associations. Future research with bigger cohorts is necessary to investigate the positive expression threshold of PD-L1 protein and its correlation with prognosis.

**Table 1:** PD-1/PD-L1 expression in TME of PCNSL and association with prognosis.

Tumor sample	Sample size	Positive definition criteria	mPD-1	Relationship with prognosis	PD-L1		Relationship with prognosis	Ref
					nPD-L1	mPD-L1		
PCNSL	n=20	mPD-1^+^: IHC-positive TIL >5%; mPD-L1/nPD-L1^+^: IHC-positive TAM/tumor cells >5%.	60%	NA	10%	20%	NA	(26)
PCNS- DLBCL	n=45	mPD-1^+^: ≥1 positive cell/HPF; mPD-L1^+^: >85% IHC-positive leukocytes (excluding tumor cells); nPD-L1^+^: >80% IHC-positive tumor cells.	67%	Irrelevant	11%	55%	Irrelevant	(27)
PCNSL	n=76	mPD-1^+^: ≥70 cells/HPF; PD-L1^+^: ≥100 cells/HPF.	46.10%	Negatively correlated with OS, PFS (P = 0.007, P = 0.028)	13.2%		Irrelevant	(28)
PCNS- DLBCL	n=98	mPD-L1^+^: ≥30% IHC-positive leukocytes (including tumor cells); nPD-L1^+^: ≥30% IHC-positive tumor cells.	NA	NA	35.70%	48%	nPD-L1 was negatively correlated with OS (P = .026); mPD-L1 was negatively correlated with PFS (P = 0.1)	(29)
PCNS- DLBCL	n=82	mPD-1^+^: ≥1 cells/HPF; PD-L1^+^: combined positivity score (CPS) ≥ 1	70.70%	Irrelevant	59.80%		Negatively correlated with PFS, OS (P = 0.001, P = 0.001)	(24)
PCNS- DLBCL	n=52	mPD-1^+^: >18.8 positive cells/mm^2^; PD-L1^+^: IHC staining intensity≥20%.	NA	Negatively correlated with percent survival (P = 0.024).	52.9%		Positively correlated with OS (P = 0.001)	(30)
PCNS- DLBCL	n=32	mPD-1^+^: IHC-positive leukocytes≥5%; PD-L1^+^: IHC-positive tumor cells≥5%.	53.1%	Negatively correlated with OS (p=0.011)	37.5%		Irrelevant	(31)

mPD-1 indicates PD-1 in the tumor microenvironment (TME) (excluding tumor cells); mPD-L1 indicates PD-L1 in TME (It is mainly PD-L1^+^TAMs, excluding tumor cells); nPD-L1 indicates PD-L1 on neoplasm cells; IHC stands for immunohistochemical staining method; HPF stands for High power field; NA stands for study did not specifically describe.

### 2.2 The role of PD-L1 protein in TME of PCNS-DLBCL

The TME of PCNS-DLBCL predominantly consists of tumor cells, diverse immune cells (including TAMs, microglia, T cells, and a limited number of B cells and DCs, etc), and stromal elements (comprising astrocytes, vascular endothelial cells, and an extracellular matrix rich in hyaluronic acid and proteoglycans). Intricate interactions occur between PD-L1 and tumor microenvironment components, eventually facilitating tumor proliferation and immune evasion. Notably, The TME of HIV^+^/EBV^+^PCNS-DLBCL displays distinct features. In contrast to immunocompetent PCNS-DLBCL, the TME of HIV^+^PCNS-DLBCL has markedly diminished levels of CD4^+^T cells and TAMs, while the EBV^+^ TME is characterized by an abundance of TAMs (32).

#### 2.2.1 Relationship between PD-L1 and TIL

**Figure 1:**
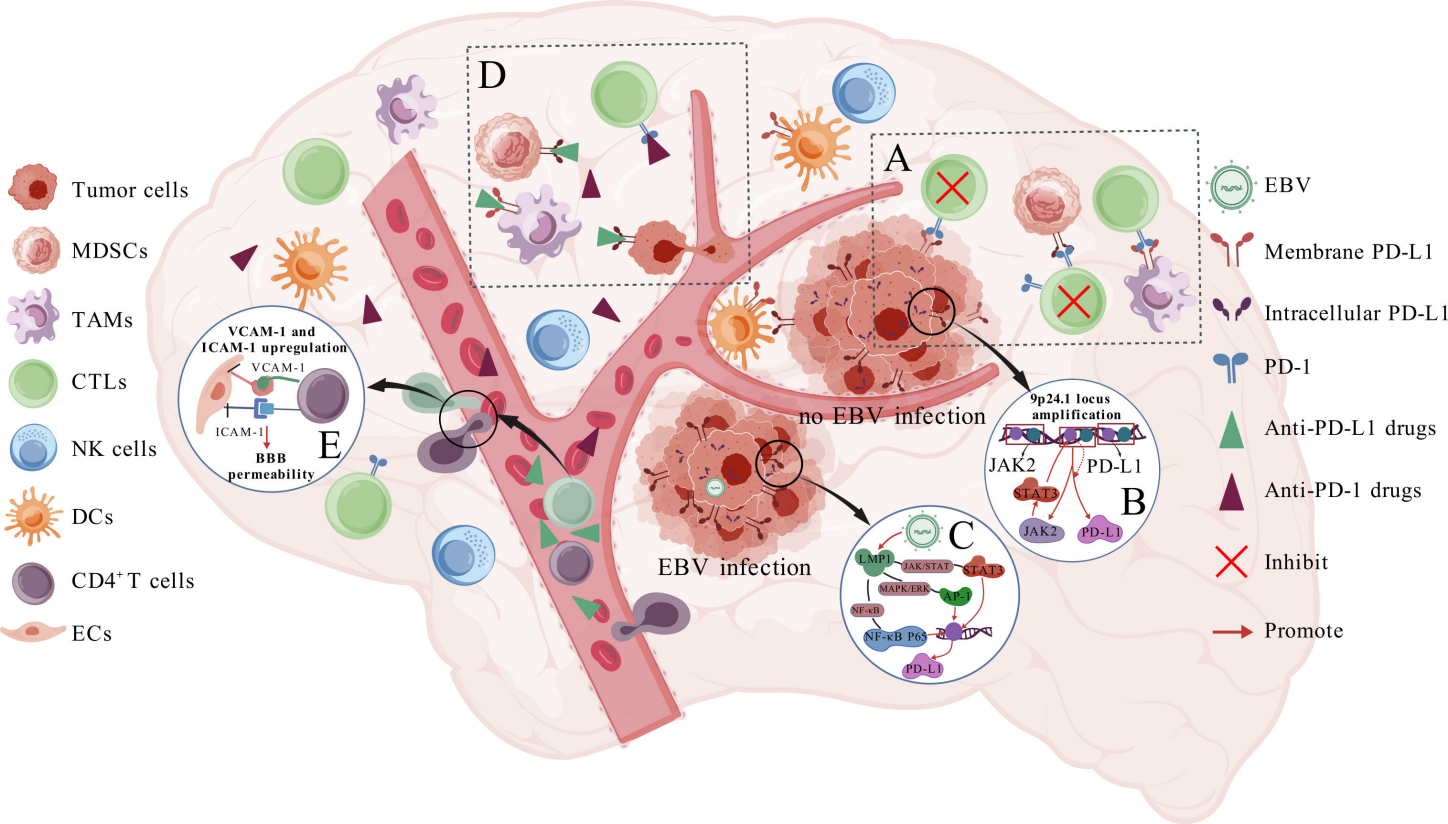
Expression and mechanism of PD-L1 protein in PCNS-DLBCL. **(A)** shows that tumor cells, TAMs, and MDSCs express PD-L1, which inhibits CTL cytotoxicity through a PD-1-dependent pathway and promotes tumor growth; **(B, C)** show how tumor cells up-regulate PD-L1 expression in cases without and with EBV infection, respectively; **(D)** shows the mechanism by which anti-PD-1/PD-L1 mAb exert their efficacy; **(E)** illustrates how CD4^+^/CD8^+^ T cells increase BBB permeability.

It has now been seen in numerous solid tumors that TILs, particularly activated CD8^+^T cells, capable of producing IFN-γ, enhancing the levels of PD-L1 in tumor cells as well as TAMs via the JAK-STAT signaling pathway. The interplay with PD-1 impedes T cell cytotoxicity, leading to Occurrence of tumor escape (**Figure 1A**) (33). Patients exhibiting PD-L1 protein overexpression in systematic DLBCL demonstrated increased CD8^+^T cells infiltration and heightened PD-1 production (34);Wada et al. didn’t identify a link between PD-L1 and PD-1 expression in newly diagnosed DLBCL; however, they noted a positive connection between mPD-1(PD-1 in the TME, excluding tumor cells) and mPD-L1 (PD-L1 in the TME, excluding tumor cells) expression in biopsy specimens from relapsed cases, showing that it may be related to the increased number of PD-L1^+^cells attracting more PD-1^+^TIL infiltration (35). In PCNSL, Marion Four et al. discovered a correlation between PD-1 levels on TILs and nPD-L1(PD-L1 on neoplasm cells) levels (P = 0.001). Overexpression of nPD-L1 may occur from significant infiltration of TILs, and the using of anti-PD-1 mAb obstructed not only inhibitory signaling but also diminished nPD-L1 expression, hence enhancing treatment response (31);However, it has been proposed that the levels of expression PD-1 on TILs correlates with mPD-L1 levels but not with nPD-L1 expression (36), Furthermore, several studies have failed to establish an association between PD-1 and PD-L1 expression (30, 37), These conflicting findings may be attributed to significant inter-tumor heterogeneity in the composition of TME in PCNSL or issues related to sample representativeness (38), Consequently, additional research is necessary to investigate this matter in the future.

#### 2.2.2 The relationship between PD-L1 and TAMs

A characteristic of immunocompetent PCNS-DLBCL TME is the extensive infiltration of TAMs (39). Furuse et al. examined tissue specimens from 70 patients with PCNS-DLBCL and determined that PD-L1 levels on peritumoral TAMs significantly predicted favorable clinical outcomes (p =0.0129) (40), However, our group (21) and several other studies reached inconsistent conclusions (**Table 1**), indicating that the correlation PD-L1 expression on tumor-associated macrophages as well as clinical outcome remains ambiguous. Additionally, the function of PD-L1 proteins on TAMs has not been thoroughly investigated. A study based on melanoma and ovarian cancer found that PD-L1 expressed on TAMs induces apoptosis in activated TILs (41); PD-L1-expressing TAMs inside the breast cancer TME augment the multiplication and cytotoxicity of CD8^+^T lymphocytes (42); In contrast, PD-L1 expression on lung cancer TAMs does not correlate with T cell responses but rather protects cells from T cell-mediated destruction (43); These observations across various solid tumors imply that the role of PD-L1 proteins on TAMs may vary by tumor type, suggesting that conclusions drawn from other tumor models may not be applicable to PCNS-DLBCL. Given the prevalence of TAMs in the microenvironment, it is vital to unravel the connection between PD-L1-expressing TAMs and the tumor immunologic response. Nevertheless, few studies have analyzed in depth the function of PD-L1 on TAMs and the infiltration-inducing relationship with PD-1^+^TIL, necessitating further investigation in future extensive studies.

#### 2.2.3 The relationship between PD-L1 and glial cells

Microglia, astrocytes, and oligodendrocytes within the tumor microenvironment of central nervous system (CNS) malignancies may also express PD-L1 (44). Microglia constitute the principal innate immune cells within the CNS (45). Studies demonstrate that in gliomas, TAMs predominantly derive from microglia (46). Therefore, microglia are essential immune cells in the TME of CNS cancers. Chauhan et al. posited that PD-L1 on microglial surfaces inhibits the synthesis of proinflammatory cytokines, including IFN-γ and TNFα, resulting in malfunction among intracranially invading T cells (44). Moreover, astrocytes diminish CD8^+^T cell activation in individuals with central nervous system infections by enhancing their own PD-L1 expression (47). These data suggest that glial cells may significantly contribute to the tumor microenvironment of PCNS-DLBCL, underscoring the need for further research in the future.

#### 2.2.4 The relationship between PD-L1 and other immune cells

Studies based on other solid tumors have shown that PD-L1 on MDSCs binds with PD-1 on T lymphocytes, thereby obstructing T lymphocytes triggering as well as cytotoxicity (48), which is one of the main mechanisms by which tumor EV-induced formation of MDSCs and M-MDSCs play a role of immunosuppression (**Figure 1A**) (49–51). Conversely, the administration of anti-PD-1/PD-L1 mAb mitigates the suppressive impact of both MDSC subpopulations. PD-L1 on melanoma-associated DCs can transmit inhibitory signals via interacting with PD-1 on T lymphocytes or via obstructing CD28 co-stimulatory signaling through cis interactions with B7.1 on its surface, thereby impeding T cell activation (52). Conversely, in a study utilizing a murine model of colorectal carcinoma, discovered that PD-L1 on DCs safeguards itself from destruction mediated by cytotoxic T cell hyperfunction. PD-L1 deficiency diminishes anti-tumor responses, and that increased PD-L1 levels in DCs is correlated with enhanced prognosis after immunochemotherapy (53). These findings imply a connection between tumor immune responses and PD-L1 expression on MDSCs and DCs. Nonetheless, no studies have yet investigated the impact of PD-L1 protein expression on MDSCs and DCs in PCNS-DLBCL on tumor immune responses, further exploration is needed in the future.

#### 2.2.5 The relationship between PD-L1 and extracellular matrix

The ECM also modulates PD-L1 expression and functionality. Hyaluronic acid (HA), a crucial component of the ECM (54), undergoes heightened synthesis, degradation, and fragmentation in pathological conditions such as malignancies. Upon detecting these changes, the receptor CD44 is activated, leading to the upregulation of PD-L1 expression in tumor cells through signaling pathways such as EGFR/Akt/mTOR, thus facilitating immune evasion (55). Moreover, research using mouse models of bladder and colon malignancies indicates that tumor-associated HA facilitates the development of PD-L1^high^M2-macrophages (56). These data suggest that the ECM likely modulates PD-L1 expression and immune activity within the tumor microenvironment of PCNS-DLBCL, warranting further investigation into its potential impact.

## 3 Molecular regulation of PD-L1 protein expression in PCNS-DLBCL

Recent gene sequencing studies, including whole exome sequencing (WES), have revealed that PCNSL exhibits a higher frequency of copy number amplification at the 9p24.1 locus compared to systemic DLBCL, and the frequency was 52% (33/63) (57). This amplification is the predominant genetic mechanism responsible for elevated PD-L1 protein levels in PCNSL neoplasm cells. In contrast, the incidence of PD-L1 overexpression caused by translocation of chromosome 9 is less than 1% (58); Furthermore, it was proposed that PD-L1 expression is an early mutational event in PCNS-DLBCL (59). The 9p24.1 amplified area includes the JAK2 locus, and JAK2 amplification increases the production and activation of the JAK2 protein, potentially facilitating increased levels of PD-L1 transcription and expression (**Figure 1B**) (60). Autophagy is regarded as a pro-tumorigenic mechanism in PCNS-DLBCL, and it has been observed that M6PR, a surface protein that aids in the translocation of target proteins to the lysosome for degradation and serves as a hallmark protein of autophagy, demonstrated a positive connection with PD-L1 expression (including nPD-L1 and mPD-L1) (61). The genetic features of PCNSL are MYD88, CD79b, and others activating the NF-kB signaling pathway (2), In other tumors, this pathway’s activation can directly enhance PD-L1 transcription, indicating a potential correlation between NF-kB signaling activation and elevated PD-L1 protein levels in PCNS-DLBCL.

Abdulla et al. found that all PCNS-DLBCL patients with high PD-L1 (n=5) and PD-L2 (n=4) expression were concurrently EBER^+^, demonstrating a notable connection between PD-L1 and EBER (27). Minderman et al. examined tumor samples from PCNSL patients (n=22) using immunohistochemical staining and fluorescence *in situ* hybridization. They found that all 3 EBV^+^ patients exhibited significant PD-L1 expression (62). Mechanistically, EBV latent membrane protein 1 (LMP1) enhances PD-L1 promoter activity, leading to high PD-L1 expression in EBV^+^ patients (**Figure 1C**). Sethi et al. only found 9p24.1 amplification in 1 of 8 PD-L1^+^EBV^+^patients(12.5%) (63), this indicates that EBV LMP1 is the primary driver of PD-L1 upregulation in EBV^+^ cases, while 9p24.1 amplification contributes less significantly in this context. In addition, a minor percentage of PCNS-DLBCL patients are infected with the HIV virus. These patients are generally younger, with more than fifty percent demonstrating concurrent EB virus infection (32). A study including 41 HIV^+^ PCNSL patients revealed detectable PD-L1 protein expression in as many as 92.7% (38/41) of instances (64). it demonstrates a strong correlation between HIV infection and the upregulation of PD-L1 expression. However, the exact mechanisms are still to be clarified. Research on various solid tumor models has found that tumor cells primarily produce PD-L1 through amplification and translocation mutations at the 9p24.1 locus, regulation of various pathways/proteins such as JAK-STAT/c-Myc/CD58 (i.e., intrinsic pathways), and induction by cytokines like IFN-γ and IL-10 (i.e., extrinsic pathways). In contrast, non-tumor cells, such as M2-like macrophages, predominantly depend on signaling molecules, such as PD-1, from tumor or peripheral immune cells to stimulate PD-L1 production and to reflect pre-existing immunity (17, 19, 29, 65, 66). However, studies that examine the regulating of PD-L1 levels of expression using PCNS-DLBCL tumor models are few. Given the distinctive blood-brain barrier architecture in PCNSL, which possesses unique attributes compared to tumors in other locations, extensive research employing PCNSL tumor models remains essential for further validation and exploration in the future.

## 4 Clinical application of anti-PD-1/PD-L1 mAb: effectiveness and biomarkers

### 4.1 Application of anti-PD-1 mAb in PCNS-DLBCL

Anti-PD-1 mAb inhibit the immunosuppressive influence of PD-L1 on T cells by specifically binding to the PD-1 receptor on T cells surfaces, hence enabling T cells to re-identify and target neoplastic cells (**Figure 1D**). Currently, Camrelizumab, Penpulimab, Sintilimab, and Tislelizumab are sanctioned by the National medical products administration (NMPA) of China for the therapy of R/R classical Hodgkin’s lymphoma (cHL)following at least second-line systemic chemotherapy, while Nivolumab is authorized in the United States Food and drug administration (FDA) for R/R cHL; Cemiplimab lacks any hematologic oncology indication at this time. This section will focus on the efficacy, mechanisms, and potential predictive biomarkers of anti-PD-1 mAb monotherapy and combination therapy.

#### 4.1.1 Efficacy of anti-PD-1 mAb monotherapy

Anti-PD-1 mAb are rarely used to treat newly diagnosed patients, these antibodies are most commonly used to induce and maintain R/R PCNS-DLBCL, treatment should persist until disease progression or the emergence of unacceptable toxicity (67). The induction treatment of R/R PCNS-DLBCL (n=4) using Nivolumab shown efficacy, with all patients achieving progression-free survival (PFS) exceeding one year (68); Nivolumab monotherapy as induction therapy achieved a 77.8% overall response rate (ORR) (44.4% PR, 33.3% CR) and a 44% 2 years overall survival (OS) rate in 9 cases with CNS-involved lymphoma (8 PCNSL, 1 testicular lymphoma) (69);Nivolumab monotherapy achieved a 40.9% objective response rate in a multicenter research (n=22) of R/R PCNS-DLBCL patients (22); Therefore, anti-PD-1 mAb display promising effectiveness in the induction of R/R patients with minimal serious adverse effects. A study analyzing soluble PD-1 levels in pretreatment cerebrospinal fluid (CSF) samples (n=11) of PCNS-DLBCL demonstrated that sPD-1 concentrations in untreated cases were elevated by 10- to 30-fold compared to non-PCNSL controls (p<0.001). Moreover, sPD-1 levels showed significant positive correlation with adverse histological features (70), providing a potential rationale for the clinical efficacy of anti-PD-1 therapy in PCNS-DLBCL.

#### 4.1.2 The effectiveness of anti-PD-1 mAb in conjunction with other therapies

##### 4.1.2.1 BTK inhibitor

In PCNSL, BTK inhibitors constitute a promising therapeutic approach, demonstrating substantial clinical benefits when employed alongside anti-PD-1 mAb. For example, in a second phase clinical study, Ibrutinib plus nivolumab induced 50% CR and 78% ORR in 18 CNS lymphoma patients (16 PCNSL, 2 secondary), with median PFS and OS of 6.6 and 25.4 months (71); In a limited number of 4 cases, induction therapy using orelabrutinib, camrelizumab, and fotemustine achieved an objective response rate (ORR) of 100% and a 6-month PFS rate of 100% (72); A rescue induction regimen utilizing a BTK inhibitor and an anti-PD-1 mAb showed enhanced efficacy, with a partial response (PR) noted after 4 months of treatment of PCNS-DLBCL (73). In terms of mechanism, BTK inhibitors, such as Ibrutinib, can significantly promote T-cell infiltration and enhance the antitumor immune response of anti-PD-1 mAb against T cells, particularly CD8^+^ memory T cells (74); Additionally, the inhibition of PD-1/PD-L1 pathway via anti-PD-1 mAb can rectify the metabolic and immune deficiencies induced by BTK inhibitors (75). Compared to CAR-T therapy, chemotherapy protocols using anti-PD-1 mAb and BTK inhibitors carry a lower risk of neurotoxicity and other side effects. Therefore, these regimens are often preferred as primary induction treatment for elderly patients unable to tolerate intensive chemotherapy. However, for patients with a good performance status who are resistant to previous multi-line therapy (even with anti-PD-1/PD-L1 antibodies), or for younger patients, CAR-T therapies may still be preferred options (76).

##### 4.1.2.2 CAR-T therapy

According to preclinical trials, Combining CAR-T (chimeric antigen receptor T cell) therapy with anti-PD-1 mAb can enhance CAR-T cell activity and promote tumor cell death (77). Clinical trials (78, 79) and individual case reports (80) have shown that The combination of Nivolumab may increase the reactions and perseverance of anti-CD19 CAR-T by reactivating PD-1^+^CRA-T and reducing themselves PD-1 expression (79). Moreover, CAR-T therapy shows effectiveness against PCNSL (pathological type is large B-cell) but has a brief remission period. This may be linked to modifications in the inflammatory infiltrate composition within the TME post-CAR-T cell therapy, including the upregulation of PD-L1 in the TME. These alterations contribute to the restricted duration of CAR-T treatment efficacy (81). Patients with PCNS-DLBCL who underwent maintenance therapy with a combination of anti-PD-1 mAb and BTK inhibitors following CAR-T treatment attained complete remission lasting more than 35 months. This suggests that the synergistic effect of anti-PD-1 mAb and BTK inhibitors reduces the overexpression of PD-L1 within the TME, thereby enhancing the therapeutic efficacy of CAR-T therapy (82). Consequently, for patients with PCNS-DLBCL who have had CAR-T treatment, combination maintenance therapy utilizing anti-PD-1 mAb and BTK inhibitors may be an excellent option.

##### 4.1.2.3 Other treatments

ACT001 is a small-molecule compound capable of crossing the blood-brain barrier to directly target brain tumor lesions. It mostly treats recurrent glioblastoma. Research suggests that ACT001 reduces PD-L1 levels of expression on PCNS-DLBCL cells, enhancing T cell anti-tumor response in a dose-dependent manner (83). Lenalidomide increases T and NK cell proliferation and activation while decreasing Treg cell activity, reducing immunosuppression (84), In PD-L1^+^PCNS-DLBCL, anti-PD-1/PD-L1 mAb can reduce immunosuppression, enhancing T cell cytotoxicity. Combining them may increase immune system activation. A phase 2 trial of Sintilimab, HD-MTX, Temozolomide, and Rituximab for newly diagnosed PCNSL showed 96.3% (25/27) ORR (85); 3 of 6 PCNS-DLBCL cases treated with rituximab and an anti-PD-1 mAb (5 Pembrolizumab, 1 Nivolumab) achieved CR (86). Wang et al. suggested that cases with R/R PCNS-DLBCL resistant to HD-MTX, Temozolomide, whole brain radiotherapy, Ibrutinib, and Lenalidomide achieved partial remission with an anti-PD-1 mAb and Thiotepa and complete remission with transplantation of autologous stem-cell. Maintenance therapy using Tislelizumab and Thiotepa occurred every three months, and patients were still in full remission after two years (87), suggesting that R/R PCNSL patients resistant to numerous lines of treatment may derive advantages from anti-PD-1 mAb. Furthermore, the safety of anti-PD-1 mAb is robust, as a Meta-study that included 7 studies found that skin responses were the most common adverse event in PCNSL cases treated with anti-PD-1 mAb (88).

### 4.2 Application of anti-PD-L1 monoclonal antibody in PCNS-DLBCL

Many recent research on PD-L1 protein show that it inhibits T cell cytotoxicity, aids neoplasms immune escape through the PD-1/PD-L1 pathway, and modulates tumor growth and proliferation through various mechanisms. anti-PD-L1 monoclonal antibody (anti-PD-L1 mAb) have presented success in several types of lymphoma (**Figure 1D**). A phase 2 trial, single-arm, multicenter of atezolizumab, venetoclax, and obinutuzumab reported a 67.9% ORR (89); The Phase 2 trial of Atezolizumab combined with Obinutuzumab and Lenalidomide for R/R follicular lymphoma (FL) (n=38) demonstrated a CR rate of 71.9% and a 36-month PFS rate of 68.4% (90); Consolidation therapy with atezolizumab significantly extended disease-free survival (DFS) and OS in DLBCL patients who achieved CR following R-CHOP chemotherapy (chemotherapy regimens containing Rituximab, Cyclophosphamide, Doxorubicin, Vincristine, Prednisone) (91, 92). In addition, phase 2 research (n=80) of the anti-PD-L1 mAb Sugemalimab for R/R extra-nodal NK/T cell lymphoma (ENKTL) displayed an 82.5% response rate within 18 months and a 44.9% ORR. Sugemalimab has been approved by the Chinese NMPA and the United States FDA to treat R/R ENKTL (93). To our knowledge, no anti-PD-L1 mAb drugs have been authorized for administering DLBCL or PCNSL. Fortunately, clinical trials investigating anti-PD-L1 mAb for PCNSL are currently in progress (Trial Approval Nos. NCT04899427, NCT04462328, NCT04688151, NCT04462328), and the results will be determined shortly.

In comparison to anti-PD-1 mAb, anti-PD-L1 mAb not only can disrupt the immunosuppressive effects of tumor cells on T cells by obstructing the PD-1/PD-L1 pathway but also partially inhibit the activity of atypical PD-L1, thereby reducing tumor growth and proliferation (94). It has to be mentioned that the anti-PD-L1 mAb Avelumab not only inhibits the PD-1/PD-L1 axis but also activates NK cell-mediated ADCC, resulting in the direct elimination of PD-L1-expressing neoplasm-cells (95). Liu et al. discovered that PD-L1 protein is also present on T-cell surfaces, where it inhibits T cell development and the excretion of critical cytokines, such as IL-2, TNF-α; however, anti-PD-L1 mAb obstruct this mechanism (96). In preclinical lung, kidney, pancreatic, and melanoma models, anti-PD-1 antibodies stimulated the PD-L1-NLRP3 inflammatory signaling in cancer-cell. The activation recruited PMN-MDSCs to the TME, causing acquired resistance in anti-PD-1 mAb patients (97). In contrast, anti-PD-L1 mAb directly target the PD-L1 protein, reducing the likelihood of resistance development. Regarding side effects, anti-PD-1 mAb impede the binding of both PD-1 protein and its other ligand, PD-L2, perhaps leading to an increased frequency of adverse events of grade 3 or higher (OR = 1.58) (98). These have displayed us the importance of anti-PD-L1 mAb in PCNSL therapeutic research and the necessity for more. Nonetheless, considering that the efficacy of anti-PD-1 versus anti-PD-L1 may also be related to different tumor types, additional studies with larger cohorts are still required to analyze this issue comparatively in the future. The subsequent table (**Table 2**) summarizes clinical trials involving anti-PD-1/PD-L1 mAb treatment protocols.

**Table 2:** Summary of clinical trials with anti-PD-1/PD-L1 mAb-containing drugs.

Clinical trial number	Phase	Target population	Treatment program
NCT04899427	2	R/R PCNSL	Sintilimab/Tislelizumab combined with Orelabrutinib induction therapy
NCT04462328	1	PCNSL	Durvalumab combined with Acalabrutinib induction therapy
NCT04688151	2/3	R/R PCNS-DLBCL	Durvalumab combined with Rituximab and Acalabrutinib induction therapy
NCT04462328	1	R/R PCNSL	Durvalumab combined with Acalabrutinib induction therapy
NCT04070040	2	Relapsed PCNS-DLBCL	Camrelizumab induction therapy
NCT02857426	2	R/R PCNSL or PTL	Nivolumab single-agent induction therapy
NCT02779101	2	R/R PCNS-DLBCL	Pembrolizumab single-agent induction therapy
NCT04845139	NA	R/R PCNS-DLBCL	Nivolumab given intrathecally induction therapy
NCT04421560	2	R/R PCNSL	Pembrolizumab combined with Ibrutinib and Rituximab induction therapy
NCT05347641	2	PCNSL	Penpulimab combined with RMA(Rituximab, Methotrexate, Cytarabine)induction therapy
NCT04831658	1/2	PCNS-DLBCL	anti-PD-1 antibody combined with Orelabrutinib and Fotemustine induction therapy
NCT04609046	1	PCNS-DLBCL	Nivolumab combined with Rituximab, Methotrexate and Lenalidomide induction therapy
NCT03770416	2	R/R PCNS-DLBCL	Nivolumab combined with Ibrutinib induction therapy
NCT03798314	1	R/R PCNS-DLBCL or PVRL-DLBCL	Nivolumab combined with Pomalidomide induction therapy
NCT03558750	1/2	R/R PCNS-DLBCL or DLBCL	Nivolumab combined with Rituximab and Lenalidomide induction therapy
NCT06556199	1b/2	R/R PCNS-DLBCL	anti-PD-1 antibody combined with Selinexor and Temozolomide induction therapy
NCT06475235	1	PCNS-DLBCL	Pembrolizumab combined with Methotrexate, Temozolomide and Rituximab induction therapy; Pembrolizumab maintenance therapy
NCT05425654	2	PCNSL	Rituximab, Methotrexate, Procarbazine, Vincristine, Lenalidomide Followed by Auto-HCT and maintenance therapy by Nivolumab
NCT04401774	2	PCNSL With ct-DNA (CSF)	Nivolumab maintenance therapy
NCT04022980	1b	PCNSL (Older Patients)	Nivolumab maintenance therapy

R/R PCNSL, Relapsed or Refractory Primary central nervous system lymphoma; PVRL-DLBCL, Primary vitreoretinal diffuse large B cell lymphoma; PTL, Primary testicular lymphoma; DLBCL, Diffuse large B cell lymphoma; CSF, Cerebrospinal Fluid; NA stands for study did not specifically describe.

### 4.3 Effectiveness biomarkers for anti-PD-1/PD-L1mAb

It is widely believed that, cases respond better to anti-PD-1/PD-L1 monoclonal antibodies when TME cells express PD-L1 (99, 100). Barfi et al. demonstrated that 4T1-breast and CT26-colon cancer cell lines responded favorably to anti-PD-L1 mAb in conjunction with Ibrutinib despite expressing minimal PD-L1 levels (101); Additionally, a patient with CD20^-^PCNSL demonstrated negative PD-L1 immunohistochemistry and FISH results, with PD-L1-expressing TAMs <10% and minimal PD-1^+^TILs. After developing resistance to HD-MTX, the patient underwent two cycles of induction therapy with zanubrutinib plus tislelizumab combination therapy and subsequently achieved complete remission, maintaining disease stability for 20 months (102). This proposes that the effectiveness of anti-PD-1 mAb cannot be accurately predicted based on a patient’s pre-treatment PD-L1 levels. Concurrently, it suggests that immunotherapy that Targeting PD-L1 may be advantageous for those who are PD-L1-negative patients as well (101). Some research explored potential predictive markers of anti-PD-1/PD-L1 mAb efficacy, as shown below:

#### 4.3.1 Tumor mutational burden

TMB possesses significant prognostic value in tumor immunotherapy; generally, In monotherapy with anti-PD-1/PD-L1 mAb, more non-synonymous TMB patients have better ORR and PFS (37). Terziev et al. discovered that a patient with PCNSL who experienced relapse following multiple autologous stem cell transplants was sustained on maintenance therapy with Nivolumab after achieving a CR for up to 3 years. WES indicated a high TMB, and IHC suggested the existence of PD-1^+^TILs, although PD-L1 IHC results were negative. Consequently, it was posited that the patient’s remission could be attributed to the high TMB and PD-1^+^TIL infiltration (103). The proposed mechanism is that a high TMB may lead to the expression of more aberrant proteins. At the same time, the peri-tumoral region is infiltrated with an increased number of TILs, which become activated upon PD-1 blockade, thereby enhancing clinical outcomes for the patient. Nonetheless, the precise threshold definition of TMB must account for factors such as the heterogeneity of treatment protocols and target demographics; furthermore, the potential for enhanced predictive accuracy through the integration of additional biomarkers, including PD-L1 levels and the quantification of TIL and TAMs, as well as the specific methodologies for such combined predictions, necessitates further investigation.

#### 4.3.2 Soluble PD-L1 and exosomal PD-L1

A study including 46 patients with PCNSL found that only the expression levels of CSF sPD-L1 significantly elevated, which demonstrated superior predictive capability for differential diagnosis and unfavorable prognosis compared to CSF sPD-L2 (104). exoPD-L1 has an identical membrane topology to mPD-L1, meaning that it also stops T-cell from activating and multiplying. Unlike mPD-L1, exoPD-L1 and sPD-L1 can circulate through the blood and lymphatic system and block PD-1 systemically (105), they also compete with anti-PD-1 mAb, reducing their efficacy. Consequently, they serve not only as indicators of PD-L1 levels of expression in the TME but are also linked to primary resistance against anti-PD-1 mAb (106), thus may predict clinical responses to anti-PD-1/PD-L1 mAb therapy.

#### 4.3.3 IFN-α

Research has shown that IFN-α can increase PD-L1 levels of expression in squamous-cell-carcinoma tumor-cell by inducing PD-L1 transcription through phosphorylated Stat1 (Tyr701) (107); Increased PD-L1 protein levels in breast neoplasm-cell maintain persistent IFN-α production through the cGAS-STING pathway, thereby promoting tumor growth (108). A phase 2 trial of Sintilimab with chemotherapy for PCNSL examined cytokines in CSF, including IL-1β, IL-2, IL-6, IL-8, IL-12P70, IL-17, TNF, IFN-α, and IFNγ. The results indicated that elevated levels of IFN-α (>1.79 pg/ml) correlated with diminished PFS post-treatment (P<0.05), suggesting that IFN-α may be a biomarker for predicting the effectiveness of PD-1/PD-L1 inhibitors. The study also found that patients with high IL-10/IL-6 ratios had worse outcomes with anti-PD-1 mAb combinations, suggesting that IL-10/IL-6 may predict efficacy (85). It is essential to recognize that, as efficacy prediction indicators for PCNSL, sensitivity and specificity should be prioritized alongside the accessibility of marker data. For instance, IL-10, IFN-α, and sPD-L1 can be acquired without tissue biopsy, making them reasonably straightforward and cost-effective, hence potentially enhancing their applicability.

## 5 Clinical challenges and future directions

### 5.1 Overcoming the blood-brain barrier

The Blood-Brain Barrier (BBB) makes it arduous to transport many medications to the peri-lesion region, and anti-PD-1 antibodies have a substantial molecular weight and rarely cross it (102, 109), which constrains therapeutic efficacy and constitutes the primary significant challenge in the management of PCNSL. In 2022, induction therapy that integrated intrathecal nivolumab with systemic chemotherapy resulted in full remission in a patient with multifocal, parenchymal recurrent PCNSL, without any associated toxicity or adverse effects, indicating that intracerebroventricular administration alone facilitates the penetration of monoclonal antibodies into the deeper brain parenchyma, thereby enhancing efficacy. Furthermore, intracerebroventricular injections utilize a smaller dosage compared to systemic administration, resulting in reduced toxicities, which is crucial for elderly patients who cannot endure high doses of systemic immunotherapy (110).

Certain researchers propose that a possible mechanism by which peripheral intravenous co-infusion of anti-PD-1/PD-L1 mAb improves brain tumor efficacy relative to standard first-line therapy is the local production of IFN-γ by CD4^+^T cells upon traversing the BBB. IFN-γ enhances the expression of vascular cell adhesion molecule 1 (VCAM-1) and intercellular adhesion molecule 1 (ICAM-1), resulting in the disruption of tight junctions between brain microvascular endothelial cells, which in turn augments BBB permeability and enables the infiltration of more lymphocytes and circulating therapeutic agents into the CNS (**Figure 1E**) (111, 112);Taggart et al. thought that anti-PD-1 mAb or CTLA-4 inhibitors activated and released CD8^+^T cells in melanoma with central and peripheral organ involvement, enhancing intracranial CD8^+^T cell trafficking via upregulation of VCAM-1and ICAM-1, leading to intracranial antitumor effects (**Figure 1E**). However, if the tumor arises in the CNS without peripheral organ involvement, intracranial tumors may evade treatment with ICIs, for example, monoclonal antibodies that target PD-1 (113), indicating that anti-PD-1/PD-L1 mAb may augment BBB permeability to a certain degree. It also reveals that we must be careful to avoid antioxidants such as Semaglutide and other drugs that downregulate VCAM-1 in treating PCNSL.

Additionally, some research has found lymphatic channels that connect the CNS to the deep cervical lymph nodes. T cells in these lymph nodes can infiltrate the cerebrospinal fluid and brain parenchyma. Additionally, both T cells and antigens located in the CNS have the ability to enter the peripheral lymphatic system, where they can activate T cells in lymphatic tissues. This may offer a perspective on circumventing the BBB (111). Recently, a novel biopolymer drug combines three components: anti-PD-1mAb, agents against c-Myc, and AP-2 to aid brain delivery. This drug has demonstrated strong brain penetration and efficacy against A20 mouse brain lymphoma by inhibiting c-Myc and the PD-1/PD-L1 pathway (114); moreover, anti-PD-L1 nanobodies (C7, 5DXW) administered locally by the adoptive cellular transfer (ACT) method demonstrated enhanced tumor infiltration and sustained efficacy in preclinical models (115), representing a successful fusion of nanotechnology and immune-targeted therapy, as well as a novel approach to surmount the BBB.

### 5.2 Promoting precision therapy with anti-PD-1/PD-L1 mAb

Patients initially negative for PD-L1 via IHC exhibited elevated PD-L1 expression upon relapse (35), indicating temporal heterogeneity in PD-L1 expression. Factors such as tumor clonal evolution, loss of CD58 surface proteins (116, 117) and the induction of chemotherapeutic agents like Temozolomide (118) may enhance PD-L1 expression, facilitating evasion of immune surveillance and resulting in standard treatment failure. Moreover, recent studies have determined that PD-L1 expression exhibits intracellular spatial heterogeneity. Currently, the predominant focus of scholarly research on PD-L1 in PCNS-DLBCL is based on IHC, which makes it difficult to distinguish membrane-bound PD-L1 on tumor-cell surfaces from atypical PD-L1 in the cytoplasm, nucleus, and other cellular areas. This represents a potential explanation for instances of elevated PD-L1 expression in immunohistochemistry while exhibiting primary resistance to anti-PD-1 mAb therapy. To sum up, the temporal and spatial heterogeneity of PD-L1 expression, coupled with the unique immune microenvironment response of PCNS-DLBCL, renders its targeted therapeutic efficacy in PCNS-DLBCL markedly variable. Future investigations must focus on elucidating the regulatory process governing PD-L1 levels of expression in PCNS-DLBCL and the interactions within the immune microenvironment, alongside multi-homology typing (CS1-4), to optimize PD-L1 protein-targeted therapy and enhance patient outcomes.

Moreover, numerous PD-L1-overexpressing tumors exhibit initial resistance to PD-1/PD-L1 monoclonal antibodies, and PCNS-DLBCL may be included. Future research is required for this cohort of patients to investigate whether primary resistance to PD-1/PD-L1 monoclonal antibodies can be surmounted and tumor eradication accomplished through combinations with other ICIs agents such as CTLA-4, LAG-3, or TIM-3 inhibitors, as well as targeting immunosuppressive cells (e.g., MDSCs, Tregs) and enhancing the expression of tumor-associated antigens, among other approaches. Furthermore, studies focused on NSCLC have demonstrated that a combination therapy involving an anti-PD-L1 mAb and anti-VEGF (e.g., bevacizumab) significantly extends PFS (119); concurrently targeting PD-L1 and other pro-tumor signaling pathways (TGFβ, CD47, VEGF, CTLA4, etc.) with bispecific antibodies (BsAbs) can yield synergistic anti-tumor effects and reduce the incidence of drug resistance (120); this approach may represent a promising therapeutic avenue for patients with PCNS-DLBCL, but the ability of this novel immunotherapy to overcome the blood-brain barrier, along with its efficacy and safety, remains an area requiring further investigation.

## 6 Summary

Approximately, 30%-60% of PCNS-DLBCL tumors have significant tumor microenvironment PD-L1 expression and 9p24.1 copy number amplification is the main mechanism of high PD-L1 expression in EBV^-^patients, and LMP1 is the main mechanism in EBV^+^PCNS-DLBCL, which promotes tumor proliferation and immune escape through PD-1-dependent or non-dependent pathways and correlates with therapeutic resistance. For PCNS-DLBCL treatment, anti-PD-1 mAb alone or alongside BTK inhibitors and chemotherapeutic medicines have showed encouraging results. Future research must focus on breaking the BBB, identifying effectiveness predictive markers, combination of immunotherapy drugs, elucidating the regulatory mechanisms of PD-L1 expression, and understanding interactions within the immune milieu to attain precision targeted and combined therapy with anti-PD-1/PD-L1 mAb in patients with PCNS-DLBCL.
